# Supplementary figures and images for: Intravascular Lithotripsy for the Treatment of Stent Underexpansion: The Multicenter IVL-DRAGON Registry
Source: J Clin Med. 2022 Mar 23;11(7):1779. doi: 10.3390/jcm11071779 (PMC9000023; doi:10.3390/jcm11071779)

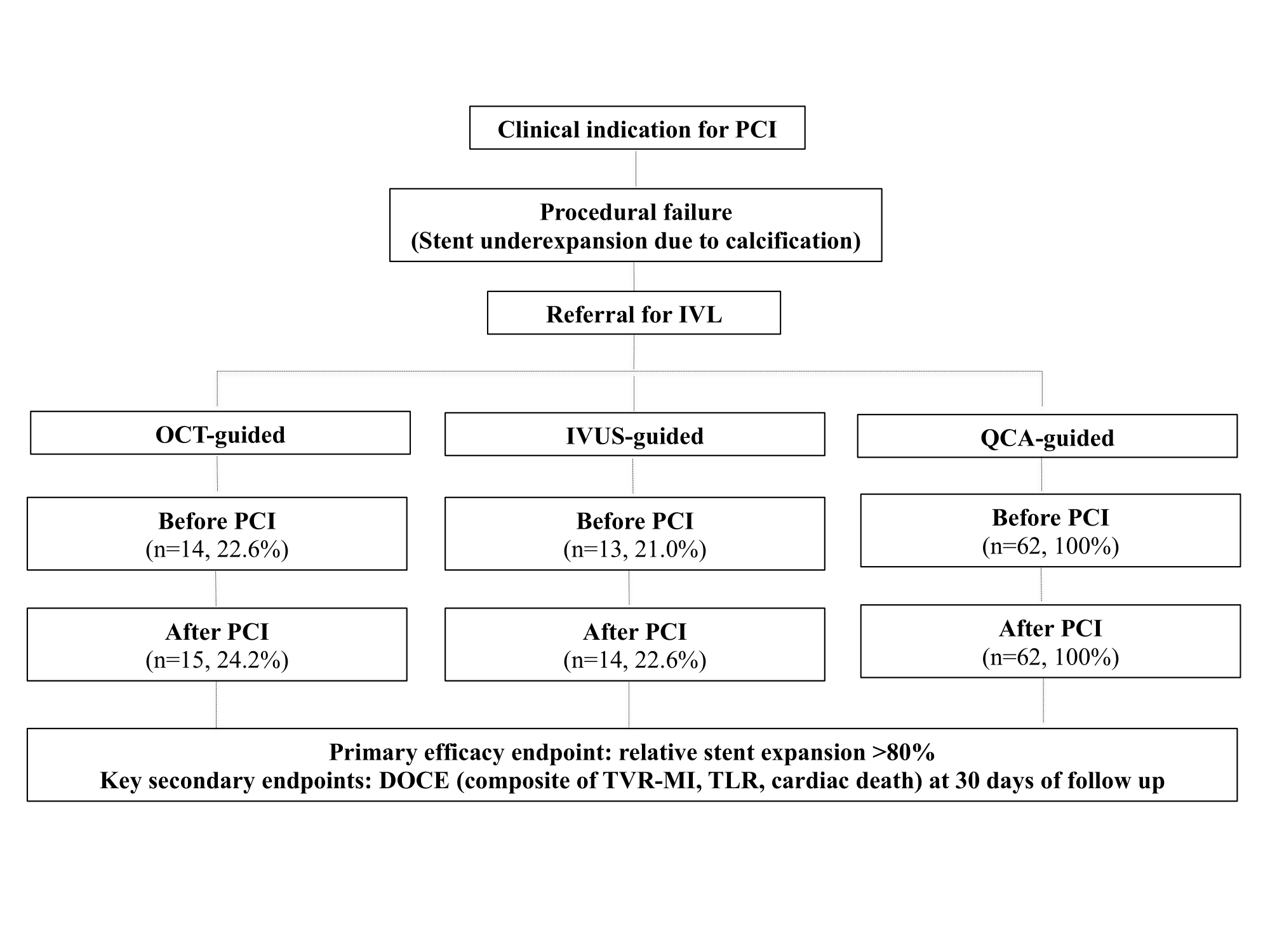

Supplement: Supplementary file 1 [file jcm-11-01779-s001.zip › jcm-1647830-supplementary Figure S1.tiff]
